# Early Mental Health and Quality of Life in Discharged Patients With COVID-19

**DOI:** 10.3389/fpubh.2021.725505

**Published:** 2021-12-23

**Authors:** Jinzhuo Hu, Yang Zhang, Qingqing Xue, Yun Song, Fei Li, Ran Lei, Jinlun Wu, Jinghua Qian

**Affiliations:** ^1^School of Sports Medicine and Rehabilitation, Beijing Sport University, Beijing, China; ^2^Rehabilitation Department, China Resources and Wisco General Hospital, Wuhan, China

**Keywords:** coronavirus disease 2019, discharged patients, mental health, quality of life, the 12-item short form Health Survey version 2

## Abstract

**Aim:** This study aimed to analyze the early mental health (MH) and quality of life (QoL) of discharged patients with coronavirus disease 2019 (COVID-19), which can provide a scientific basis for the further development of intervention programs.

**Methods:** In total, 108 subjects participated in this study, including an experimental group (90 patients diagnosed with COVID-19 from March to April 2020 and hospitalized in Wuhan China Resources & WISCO General Hospital, Wuhan, China, 83.3%) and a control group (18 healthy participants, 16.7%). Their MH and QoL were measured through the 12-item Short Form Health Survey version 2 (SF-12v2), the Self-rating anxiety scale (SAS), the Self-rating depression scale (SDS), and the International Physical Activity Questionnaire (IPAQ). The results of questionnaires were compared between these two groups.

**Results:** (1) Comparison of anxiety status: among 90 discharged patients with COVID-19, 30 patients (33.3%) had a state of anxiety. Compared with healthy participants and the general population, patients with COVID-19 in the early stages of discharge had a higher incidence of anxiety and more severe anxiety symptoms (*P* < 0.05). (2) Comparison of depression status: among 90 discharged patients with COVID-19, 29 patients (32.2%) had a state of depression. Compared with healthy participants and the general population, patients with COVID-19 in the early stages of discharge had a higher incidence of depression and more severe depression symptoms (*P* < 0.05). (3) Comparison of QoL: 78 patients (86.7%) presented a decrease in physical health-related quality of life (HRQoL) and 73 patients (81.1%) presented a decrease in psychology-related QoL. The SF-12v2 physical component summary (PCS) and the SF-12v2 mental component summary (MCS) of patients were significantly lower than those of healthy people, especially in physical function (PF), vitality (VT), social function (SF), and mental health (MH) (all *P* < 0.05). (4) Gender differences in mental health and the QoL among patients with COVID-19: women had more severe anxiety/depression symptoms than men (*P* < 0.05). The scores of women in all dimensions of SF-12V2 were lower than those of men, and there were statistically significant differences between the two groups in PCS, PF, general health (GH), VT, and role-emotional (RE) (*P* < 0.05).

**Conclusion:** During the early phase after being discharged, patients with COVID-19 might experience negative emotions, such as anxiety or depression, and also problems with reduced QoL, especially among female patients. Therefore, an intervention plan should focus on strengthening psychological condition and improving physical function, and gender-specific rehabilitation programmes should be adapted to improve psychological status and QoL.

## Introduction

An outbreak of pneumonia of unknown cause occurred in Wuhan, Hubei Province, China, in December 2019. On February 11, 2020, the WHO officially named the pneumonia caused by the novel coronavirus as coronavirus disease 2019 (COVID-19) (1). Subsequently, many people in other countries worldwide were found to be infected with the respiratory infectious disease. As of March 31, 2020, COVID-19 had caused 862,234 confirmed infection cases and 42,424 deaths, posing an important threat to the lives and health of the global population (2). The main clinical characteristics of COVID-19 are fever, cough, and shortness of breath, and a proportion of patients may also suffer from new loss of taste or smell, diarrhea, nausea, vomiting, and other symptoms (3). As the most severely affected city in Hubei Province, the health consequences of these patients with COVID-19 have attracted worldwide attention and need to be evaluated urgently.

Due to isolation and lack of awareness of the consequences of the novel coronavirus, patients with COVID-19 have been under tremendous psychological pressure during the treatment against severe acute syndrome coronavirus 2 (SARS-CoV-2), which may bring them certain mental health problems, such as anxiety, depression, insomnia, and fear (4). A recent meta-analysis included 62 studies from 17 countries and found that the prevalence of anxiety and depression was the highest (56 and 55%) among patients with COVID-19 (5). Such mental health concerns may lead to both shorter- and longer-term problems, particularly when experienced in combination with other factors, such as poverty and insufficient healthcare services (6). However, the infectiousness of COVID-19 makes it difficult for researchers to reach patients directly and continuously. Most epidemic-related psychological studies are mainly concerned with ordinary residents and medical staff (7), while there are very few follow-up investigations of mental health among discharged patients and these are rarely compared with patients who were not infected with COVID-19.

In recent years, health-related quality of life (HRQoL) has aroused great interest among researchers. Studies have shown that COVID-19 can affect HRQoL of patients and general populations (8). The patients with COVID-19 who were admitted to hospitals during infection had a low QoL score in physical, psychological, and socials domains, and continued to have QoL issues even after recovery (9). However, only few studies have reported HRQoL of patients after discharge. One study has revealed that COVID-19 is associated with a substantial and measurable decrease in HRQoL, and the age and hospitalization status of participants were the key determinants of their COVID-19 health utility value (10). Further evidence suggests that, even though physical function was recovered, patients might still have mental disorders (11), which could affect them even after 1 year (12). Therefore, the impact of SARS-CoV-2 on the psychology and HRQoL of patients cannot be ignored.

Considering studies reporting the physical and psychological conditions of patients with COVID-19 after discharge from hospital are rare, the purpose of this study is to investigate the early psychology and QoL of clinically cured and discharged patients with COVID-19 in Wuhan, a city heavily affected by novel coronavirus 2019, so as to provide a basis for further scientific intervention plans.

## Methods

### Subjects and Study Design

In total, 108 participants with and without COVID-19 were recruited in China Resources & WISCO General Hospital from March to April 2020, including an experimental group (90 patients diagnosed with COVID-19, 83.3%) and a control group (18 healthy participants, 16.7%). The inclusion and exclusion criteria of the experimental group are presented in Table 1. The control group recruited healthy people who were not infected with COVID-19 from healthcare workers and families of patients. The age and gender of the control group matched those of the experimental group, and the exclusion criteria were the same as those of the experimental group. Basic information of the two groups was collected, such as age, sex, disease type, educational status, and comorbidities of patients with COVID-19.

**Table 1:** Article inclusion and exclusion criteria.

**Inclusion criteria**	**Exclusion criteria**
(1) A clear diagnosis of COVID-19	(1) Temperature >38°C
(2) Within 3–45 days after discharge (including patients in isolation sites after discharge and early home-based patients who have returned to the community)	(2) Heart rate > 120 bpm or <40 bpm, systolic blood pressure <90 mmHg or > 180 mmHg, respiratory rate >25 bpm or vital signs significant fluctuations calm state
(3) 18–80 years old	(3) Continuous oxygen therapy was needed
(4) Voluntarily agreed to join the queue and cooperate with the relevant assessments	(4) Patients with myocarditis, pulmonary hypertension, congestive heart failure, acute renal failure, fresh venous thromboembolic disease, unstable fracture, etc., who were not suitable for exercise
	(5) Conscious disorders, cognitive dysfunction, mental disorders, balance disorders, severe bone and joint diseases, and other impact assessment patients
	(6) Other conditions of inability to cooperate with rehabilitation treatment

The mental health and Qol of all participants were measured through the 12-item Short Form Health Survey version 2 (SF-12v2), the Self-rating anxiety scale (SAS), and the Self-rating depression scale (SDS). The questionnaires were completed online and distributed in a WeChat group. The same IP address can be used only once and the questionnaire must be completed by the subject. Repeated questionnaires were eliminated. The obtained data were input into the “Questionnaire Star” system for real-time monitoring to ensure the accuracy of the data. All subjects signed the informed consent form.

### Self-Rating Anxiety Scale

The Self-rating anxiety scale is a widely used self-rating tool for adults with anxiety symptoms. The scale consists of 20 items, such as forward score and reverse score, and each item is scored at four levels. After the self-assessment, the total score was multiplied by 1.25 to get an integer. The higher the final score, the more severe the symptoms (13). According to the results of the healthy Chinese population, the cut-off value of SAS SD is 50 points, in which 50–59 is considered mild anxiety, 60–69 is considered moderate anxiety, and 69 or above is considered severe anxiety. The results need to be compared with the Chinese norm, which can provide a baseline for interpretation and comparison of the results (14).

### Self-Rating Depression Scale

The Self-rating depression scale can effectively reflect the symptoms of depression and its severity and changes. The scale consists of 20 items, each of which corresponds to one symptom concerned, and is rated on a scale of 1–4. The raw score can be converted to an SDS Index score by multiplying the raw score by 1.25. According to the results of the Chinese norm, the cut-off value of SDS standard score is 53 points, of which 53–62 is considered mild depression, 63–72 is considered moderate depression, and 73 or above is considered severe depression (15). The results need to be compared with the Chinese norm, which can provide a baseline for interpretation and comparison of the results.

### The 12-Item Short Form Health Survey Version 2

Studies have proved the applicability of SF-12v2 in the Chinese population (16). The SF-12v2 scale has 12 items, evaluating eight dimensions of HRQoL, such as general health (GH), physical functioning (PF), role-physical (RP), bodily pain (BP), vitality (VT), social functioning (SF), role-emotional (RE), and mental health (MH). GH, PF, RP, and BP can be calculated to obtain the physical component summary (PCS), while SF, RE, MH, and VT can be calculated to get the mental component summary (MCS). In the scoring calculation, corresponding weights should be given to each item according to the degree of impact on QoL (17) (Table 2). Each dimension should be converted into a percentage system, and the total physical health score and the total mental health standard score should be converted into normal-based score according to the standard where the mean is 50 and the SD is 10. A total score of more than 50 on the SF-12v2 scale indicates that the QoL is higher than that of the general population, while a score below 50 indicates that the QoL is lower than that of the general population.

**Table 2:** Conversion table of each dimension of the 12-item Short Form Health Survey version 2 (SF-12v2).

**Scale**	**Items**	**Score ranges**	**Conversion points**
GH	1	1–5	(Actual score-1)/4*100
PF	2	2–6	(Actual score-2)/4*100
RP	2	2–10	(Actual score-2)/8*100
RE	2	2–10	(Actual score-2)/8*100
BP	1	1–5	(Actual score-1)/4*100
VT	1	1–5	(Actual score-1)/4*100
MH	2	2–10	(Actual score-2)/8*100
SF	1	1–5	(Actual score-1)/4*100

### Statistical Analysis

All valid data were entered into Excel 2016 after review, sorting, and coding. SPSS 25.0 software was used for statistical analysis. Descriptive analysis was conducted on the basic information of the research subjects. Age presented a normal distribution, reported by means ± SD. *T*-test was used for comparison between groups; qualitative data were presented as the number of cases (%), and *x*^2^ test was used for comparison between the groups. Anxiety, depression, and QoL were compared between the patients with COVID-19 and healthy subjects using independent sample *t*-test and Wilcoxon signed-rank test. *P*-values < 0.05 were considered statistically significant.

## Results

In total, 111 questionnaires were collected, of which 108 were valid, with an effective rate of 97.3%.

### Characteristics of Subjects

According to the questionnaire response, there were 108 subjects in this study, including 90 (83.3%) patients with COVID-19 and 18 (16.7%) healthy participants. Among the patients with COVID-19, the mean age was (50.8 years ± 12.5) including 40 men (44.4%) and 50 women (55.6%). In this study, 9 (10.0%) patients were mild type, 63 (70.0%) patients were ordinary type, 18 (20.0%) patients were severe type, but there were no critically ill patients. All the patients were cured and discharged. In addition, we examined the education status and comorbidities of patients with COVID-19, with 37 (41.1%) having a college education or above, 28 (31.1%) having a senior high school education, and 25 (27.8%) having a junior high school education or below, and 47 (52.2%) having comorbidities (such as diabetes and hypertension). In the healthy participants, the mean age was (51.3 years ± 12.5), including nine men (50.0%), nine women (50.0%), and five participants (27.8%) with comorbidities. There was no statistical difference in general data between the two groups (*p* > 0.05) (Table 3).

**Table 3:** The baseline characteristics of patients with COVID-19 and healthy participants.

	**COVID-19 patients**	**Healthy participants**	***t/χ^2^***	***P*-value**
	***n* = 90 (%)**	***n* = 18 (%)**		
Age-years (Mean ± SD)	(50.8 ± 12.5)	(51.3 ± 12.5)	−0.165	0.869
**Gender**				
Male	40 (44.4%)	9 (50.0%)	0.187	0.666
Female	50 (55.6%)	9 (50.0%)		
**Clinical typing**				
Mild	9 (10.0%)			
Ordinary	63 (70.0%)			
Severe	18 (20.0%)			
**Educational status**				
University and above	37 (41.1%)			
Senior high school	28 (31.1%)			
Junior high school and below	25 (27.8%)			
**Comorbidities**				
Yes	47 (52.2%)	5 (27.8%)	3.590	0.058
No	43 (47.8%)	13 (72.2%)		

### Comparison of Anxiety With or Without COVID-19

Among patients with COVID-19, 30 (33.3%) had anxiety symptoms, of which 20 (22.2%) had mild anxiety, 7 (7.8%) had moderate anxiety, and 3 (3.3%) had severe anxiety. Among the healthy participants, 5 (27.8%) had anxiety symptoms, including 4 (22.2%) with mild anxiety and 1 (5.6%) with moderate anxiety. There was no significant difference in the frequency ratio of anxiety between the two groups although there was a difference in the raw scores (*P* > 0.05) (Table 4). Compared with healthy people, patients with COVID-19 had a significantly higher SAS score (*P* < 0.05). In addition, the anxiety levels in patients with COVID-19 were higher than the general population (*P* < 0.001) (Table 5).

**Table 4:** Incidence of anxiety reported by coronavirus disease 2019 (COVID-19) survivors and healthy participants.

	**Self-rating anxiety scale (SAS)**					
	**Mild**	**Moderate**	**Severe**	**None**	***χ^2^***	***P*-value**
COVID-19 patients (*n* = 90)	20 (22.2%)	7 (7.8%)	3 (3.3%)	60 (66.7%)	0.211	0.646
Healthy participants (*n* = 18)	4 (22.2%)	1 (5.6%)	0 (0)	13 (72.2%)		

**Table 5:** Average score of the Self-rating anxiety scale (SAS) reported by COVID-19 survivors, healthy participants, and the general population.

	**COVID-19 patients (*n* = 90) mean (SD)**	**Healthy participants (*n* = 18) mean (SD)**	**Mean difference between groups (95% CI)**	***P*-value**	**Effect size**
SAS score	45.72 (10.79)	39.72 (11.87)	6.00 (0.38–11.62)	0.036	0.52
	**COVID-19 patients (*****n*** **=** **90) mean (SD)**	**General population (*****n*** **=** **2,249) mean (SD)**	**Mean difference between groups (95% CI)**	***P*** **-value**	**Effect size**
SAS score	45.72 (10.79)	29.78 (0.46)	15.94 (15.49–16.39)	<0.001	2.09

### Comparison of Depression With or Without COVID-19

Among patients with COVID-19, 29 (32.2%) had depressive symptoms, of which 23 (25.5%) had mild depression and 6 (6.7%) had moderate depression. Among healthy participants, three people (16.7%) had mild depression. There was no significant difference in the frequency ratio of depression between the two groups although there was a difference in the raw scores (*P* > 0.05) (Table 6). Compared with healthy participants, patients with COVID-19 had a significantly higher SDS score (*P* < 0.05). In addition, the depression levels in patients with COVID-19 were higher than the general population (*P* < 0.001) (Table 7).

**Table 6:** Incidence of depression reported by COVID-19 survivors and healthy participants.

	**Self-rating depression scale (SDS)**					
	**Mild**	**Moderate**	**Severe**	**None**	***χ^2^***	***P*-value**
COVID-19 patients (*n* = 90)	23 (25.5%)	6 (6.7%)	0 (0)	61 (67.8%)	1.741	0.187
Healthy participants (*n* = 18)	3 (16.7%)	0 (0%)	0 (0)	15 (83.3%)		

**Table 7:** Average score of the Self-rating depression scale (SDS) reported by COVID-19 survivors, healthy participants, and the general population.

	**COVID-19 patients (*n* = 90) mean (SD)**	**Healthy participants (*n* = 18) mean (SD)**	**Mean difference between groups (95% CI)**	***P*-value**	**Effect size**
SDS score	47.28 (9.93)	41.94 (11.74)	5.34 (0.09–10.58)	0.046	0.49
	**COVID-19 patients (*****n*** **=** **90) mean (SD)**	**General population (*****n*** **=** **2,249) mean (SD)**	**Mean difference between groups (95% CI)**	***P*** **-value**	**Effect size**
SDS score	47.28 (9.93)	41.88 (10.75)	5.40 (3.14–7.66)	<0.001	0.52

### Comparison of QoL With or Without COVID-19

The scores of patients with COVID-19 and healthy participants in eight dimensions are shown in Table 8. Compared with the healthy participants, the scores of all dimensions of patients with COVID-19 were lower, and there were significant differences between the two groups in PF, VT, SF, and MH (*P* < 0.05).

**Table 8:** Average score of SF-12v2 components reported by COVID-19 survivors and healthy participants.

**SF-12v2 component**	**COVID-19 patients (*n* = 90) mean (SD)**	**Healthy participants (*n* = 18) mean (SD)**	**Mean difference between groups (95% CI)**	***P*-value**	**Effect size**
PF	73.89 (26.21)	93.06 (11.52)	−19.17 (−31.69 to −6.65)	0.003	−0.95
RP	58.06 (27.94)	67.36 (27.50)	−9.30 (−23.57 to 4.96)	0.199	
BP	55.83 (29.04)	69.44 (26.51)	−13.61 (−28.28 to 1.05)	0.069	
GH	45.28 (27.00)	55.56 (29.15)	−10.28 (−24.28 to 3.72)	0.149	
VT	58.89 (26.05)	73.61 (13.48)	−14.72 (−27.25 to −2.20)	0.022	−0.71
SF	36.94 (32.72)	75.00 (29.70)	−38.06 (−54.57 to −21.54)	<0.001	−1.22
RE	62.92 (24.96)	72.92 (24.72)	−10.00 (−22.76 to 2.76)	0.123	
MH	65.28 (21.02)	76.39 (15.39)	−11.11 (−21.46 to −0.76)	0.036	−0.60
PCS	37.85 (12.63)	46.56 (9.63)	−8.71 (−14.96 to −2.47)	0.007	−0.78
MCS	38.81 (13.54)	46.56 (11.90)	−7.75 (−14.55 to −0.94)	0.026	−0.61

The average PCS score of patients with COVID-19 was (37.85 ± 12.63), of which 86.7% of patients (78 patients) scored <50 points. The average score of MCS was (38.81 ± 13.54), and 81.1% of the patients (73 patients) scored <50 points. The statistical results showed that the scores of the patients in both physiological and psychological fields were significantly lower than those of the healthy participants (*P* < 0.05).

### Gender Differences in MH and the QoL Among Patients With COVID-19

Gender differences in MH and QoL among patients with COVID-19 are shown in Table 9. Women had more severe anxiety/depression symptoms than men (*P* < 0.05). The scores of women in all dimensions of SF-12v2 were lower than those of men, and there were statistically significant differences between the two groups in PCS, PF, GH, VT, and RE (*P* < 0.05).

**Table 9:** Gender differences in mental health (MH) and the quality of life (QoL) among COVID-19 survivors.

**Outcome variable**	**Men (*N* = 40)**	**Women (*N* = 50)**	**Mean difference between groups**	***P*-value**	**Effect size**
	**mean (SD)**	**mean (SD)**	**(95% CI)**		
SAS	41.81 (8.41)	48.85 (11.52)	−7.04 (−11.36 to −2.71)	0.002	−0.70
SDS	44.97 (9.01)	49.13 (10.33)	−4.16 (−8.27 to −0.04)	0.048	−0.43
PCS	41.03 (10.51)	35.30 (13.68)	5.73 (0.52 to 10.95)	0.032	0.47
MCS	41.26 (12.58)	36.86 (14.08)	4.40 (−1.27 to 10.06)	0.127	
PF	81.88 (21.17)	67.50 (28.23)	14.38 (3.69 to 25.06)	0.009	0.58
RP	62.50 (25.63)	54.50 (29.42)	8.00 (−3.72 to 19.72)	0.179	
BP	62.50 (28.87)	50.50 (28.34)	12.00 (−0.05 to 24.05)	0.051	
GH	52.50 (27.62)	39.50 (25.30)	13.00 (1.89 to 24.11)	0.022	0.49
VT	66.25 (23.72)	53.00 (26.55)	13.25 (2.57 to 23.93)	0.016	0.53
SF	37.50 (32.52)	36.50 (33.20)	1.00 (−12.87 to 14.87)	0.886	
RE	69.06 (20.01)	58.00 (27.52)	11.06 (0.74 to 21.38)	0.036	0.46
MH	70.00 (61.50)	61.50 (20.80)	8.50 (−0.23 to 17.23)	0.056	

## Discussion

In the face of newly emerging infectious diseases, the incidence of negative emotions, such as fear, sadness, and tension among the population increases (18). This study found that more than one-third of patients with COVID-19 had anxiety/depression. A meta-analysis also found similar results (11). This indicates that due to the long period of isolation and treatment, patients will have a sense of social alienation, anxiety, fear, and even pessimistic attitude about returning to society. However, although the incidence of COVID-19 anxiety/depression was higher than that of healthy people, the difference between the two groups was not statistically significant, which was considered to be related to the small sample size. In addition, because MH and psychosocial consequences of COVID-19 has a serious impact on various categories of people, the anxiety/depression incidence of healthy people may also increase (19).

The anxiety and depression levels in patients with COVID-19 were higher than the general population in this study. This suggests that a patient with COVID-19 may be more likely have severe anxiety/depression symptoms. In addition, we also found that the anxiety and depression of patients with COVID-19 are generally more severe than those of healthy people. This can indicate that the psychological problems of patients with COVID-19 are caused by COVID-19 infection. In addition, the difference between the two groups was small, which suggested that the COVID-19 pandemic also resulted in challenges for healthy people.

It should be noted that the baseline difference in comorbidities between the experimental group and the control group was nearly significant (*P* = 0.058). This suggests that comorbidities may have an impact on the mental status of patients with COVID-19. Analyzing the clinical and epidemiological data of COVID-19 suggested that specific comorbidities increase the risk of infection with worse lung injury and death. The most common comorbidities reported up until now were hypertension, cardiovascular diseases, and diabetes (20). Additionally, a high proportion of patients with COVID-19 and other conditions in admitted intensive care unit (ICU) cases suggested comorbidities as a potential risk factor for patients with COVID-19 (21). Therefore, the meticulous management of patients with COVID-19 with comorbidities in contrast to without comorbidities is emphasized to control the jeopardy of life. Comorbid individuals must undertake vigilant preventive measures to protect themselves during the pandemic (22).

Evidence from the present study indicated that compared with the healthy population, patients with COVID-19 had lower SF-12v2 scores in all dimensions at the early stage of discharge, especially in PF, VT, SF, MH, PCS, and MCS. These six aspects indicated that the QoL of patients was generally reduced in the early stage after discharge. Individual level variables of COVID-19 anxiety and personal identity significantly predicted QoL (23). A sense of coherence as a marker of QoL may be considered as a psychological process influencing MH, which in turn may affect QoL as well (24).

Studies have shown that gender was associated with MH and QoL for patients with COVID-19 (25, 26). Our study found that women have more severe psychological symptoms than men, which significantly affect their QoL. Findings from epidemiological studies indicate that women are at higher risk of psychological outcomes (27). Some researchers hypothesize that part of the increase in psychological stress among women may be due to their work being more affected by COVID-19 and the burden of home care (28, 29). Sex differences in self-reported stress are further reflected in the perceived need of psychological support services, which are often most evident in women (25). These findings call for active rehabilitation of patients with COVID-19 and highlight the difference in recovery between men and women.

Additionally, a reduction in physical activity participation is known to contribute to stress levels, which is strongly associated with QoL. Appropriate exercises (e.g., strength training, walking, lifting, and Qigong) are recommended behavioral strategies to promote the overall health of people (30). Exercise rehabilitation can enhance immune function, reduce the risk of infection, improve the prognosis, QoL, and the activity of daily living (31, 32). It is particularly emphasized that Qigong can relieve psychological stress, depression, and anxiety, and improve sleep quality (33).

In this study, the mental status of patients with COVID-19 is significantly reduced compared to that of the healthy participants. However, due to the small number of people in the control group, it is not possible to provide a more effective comparison to determine whether COVID-19 is the cause of mental status problems. Therefore, large sample size and high-quality randomized controlled studies should be conducted in the future. In this paper, healthy people were selected as the control group to explore the psychological status and QoL of post-discharge patients with COVID-19. However, to better exclude the impact of hospitalization on mental status, the hospitalized patients with other diseases could also be selected as the control group. Another limitation is that self-reported tools of anxiety and depression may not always be aligned with assessment by MH professionals. Our study used SAS and SDS to measure symptoms of anxiety and depression, which are different from a clinical diagnosis and cannot measure severe psychiatric symptoms, such as suicidal ideation or psychotic experience. Finally, this study cannot reveal causality. Large-scale prospective, longitudinal studies are recommended to better describe the predictors of psychological disorders and QoL in patients with COVID-19.

## Conclusions

Patients with COVID-19 have negative emotions, such as anxiety or depression and problems related to physical or psychological QoL in the early stage after discharge. Considering the negative impact of depression and anxiety on daily life and health outcomes, timely screening and appropriate interventions, such as online psychological counseling tailored for concerns specific to different genders, especially female patients, are urgently needed to reduce the likelihood of emotional disturbances after discharge. Meanwhile, patients should insist on rehabilitation training to improve their physical function and thus improve their QoL.

## Data Availability Statement

The original contributions presented in the study are included in the article/supplementary material, further inquiries can be directed to the corresponding author/s.

## Ethics Statement

The studies involving human participants were reviewed and approved by the Medical Ethics Committee of China Resources & Wisco General Hospital. The patients/participants provided their written informed consent to participate in this study.

## Author Contributions

JH, YZ, and JQ: conceptualization and writing-original draft preparation. JH, QX, YS, FL, RL, and JW: methodology, data curation, and investigation. JH, YZ, and JQ: writing-review and editing. JQ: supervision, funding acquisition, and project administration. All authors contributed to the article and approved the submitted version.

## Funding

This research was supported by a grant from the Fundamental Research Funds for the Central Universities (grant number: 2020091).

## Conflict of Interest

The authors declare that the research was conducted in the absence of any commercial or financial relationships that could be construed as a potential conflict of interest.

## Publisher's Note

All claims expressed in this article are solely those of the authors and do not necessarily represent those of their affiliated organizations, or those of the publisher, the editors and the reviewers. Any product that may be evaluated in this article, or claim that may be made by its manufacturer, is not guaranteed or endorsed by the publisher.
